# Role of Free Radicals and Biotransformation in Trichloronitrobenzene-Induced Nephrotoxicity In Vitro

**DOI:** 10.3390/ijms18061165

**Published:** 2017-05-31

**Authors:** Gary O. Rankin, Connor Tyree, Deborah Pope, Jordan Tate, Christopher Racine, Dianne K. Anestis, Kathleen C. Brown, Mason Dial, Monica A. Valentovic

**Affiliations:** Department of Biomedical Sciences, Joan C. Edwards School of Medicine, Marshall University, Huntington, WV 25755, USA; tyree31@live.marshall.edu (C.T.); pope26@live.marshall.edu (D.P.); tatej@marshall.edu (J.T.); racine@live.marshall.edu (C.R.); anestis@marshall.edu (D.A.K.); brown364@marshall.edu (K.C.B.); dial40@live.marshall.edu (M.D.); valentov@marshall.edu (M.A.V.)

**Keywords:** trichloronitrobenzenes, nephrotoxicity, in vitro, antioxidants, biotransformation

## Abstract

This study determined the comparative nephrotoxic potential of four trichloronitrobenzenes (TCNBs) (2,3,4-; 2,4,5-; 2,4,6-; and 3,4,5-TCNB) and explored the effects of antioxidants and biotransformation inhibitors on TCNB-induced cytotoxicity in isolated renal cortical cells (IRCC) from male Fischer 344 rats. IRCC were incubated with a TCNB up to 1.0 mM for 15–120 min. Pretreatment with an antioxidant or cytochrome P450 (CYP), flavin monooxygenase (FMO), or peroxidase inhibitor was used in some experiments. Among the four TCNBs, the order of decreasing nephrotoxic potential was approximately 3,4,5- > 2,4,6- > 2,3,4- > 2,4,5-TCNB. The four TCNBs exhibited a similar profile of attenuation of cytotoxicity in response to antioxidant pretreatments. 2,3,4- and 3,4,5-TCNB cytotoxicity was attenuated by most of the biotransformation inhibitors tested, 2,4,5-TCNB cytotoxicity was only inhibited by isoniazid (CYP 2E1 inhibitor), and 2,4,6-TCNB-induced cytotoxicity was inhibited by one CYP inhibitor, one FMO inhibitor, and one peroxidase inhibitor. All of the CYP specific inhibitors tested offered some attenuation of 3,4,5-TCNB cytotoxicity. These results indicate that 3,4,5-TCNB is the most potent nephrotoxicant, free radicals play a role in the TCNB cytotoxicity, and the role of biotransformation in TCNB nephrotoxicity in vitro is variable and dependent on the position of the chloro groups.

## 1. Introduction

Chloronitrobenzenes are widely used as chemical intermediates in the production of dyes, pesticides, drugs, and other commercial products [1,2,3,4,5]. Exposure to these compounds occurs primarily in an occupational setting, but chloronitrobenzenes are also present in the environment from wastewater at industrial sites and from accidental spills [6,7,8].

Information concerning the potential toxicity induced by the chloronitrobenzenes comes primarily from studies on monochloronitrobenzenes (MCNBs). These compounds induce hematotoxicity, hepatotoxicity, immunotoxicity, splenotoxicity, and nephrotoxicity [9,10,11,12,13,14,15]. Hematotoxicity is generally the most common toxicity induced by MCNBs and is present as methemoglobinemia and anemia, and these conditions may contribute to toxicity in other organs [9,10,11]. Although the toxicity induced by MCNBs has been studied to some degree, few studies have examined the toxicity induced by higher chlorinated nitrobenzenes (dichloro-, trichloro-, etc.), and it is generally believed that dichloronitrobenzenes (DCNBs) and trichloronitrobenzenes (TCNBs) have the potential to induce the same kinds of toxicities as the MCNBs.

While there have been several studies examining chloronitrobenzene-induced hematoxicity, few studies have explored the nephrotoxicity induced by these compounds. Yoshida et al. found that 4-chloronitrobenzene (1.0 mmol/kg, intraperitoneally) induced diuresis, increased *N*-acetyl-β-d-glucosaminidase excretion, and caused swelling of the kidney 48 h after treatment [14]. However, urinary creatinine and blood urea nitrogen (BUN) concentrations were not altered in the 4-chloronitrobenzene treatment group, suggesting that only a mild nephrotoxicity was induced. Matsumoto et al. [13] found that feeding 2-chloronitrobenzene to Fisher 344 rats increased the number of renal cell adenomas in females and renal carcinomas in males and exacerbated age-related chronic progressive nephropathy in males, leading to the death of 47 of 50 rats treated at the 2000 ppm level. Hong et al. found that pyruvate-stimulated gluconeogenesis was significantly reduced at 120 min in renal cortical slices from male Fischer 344 rats incubated with 1.0 mM of any of the three MCNBs [12]. However, lactate dehydrogenase (LDH) release, a marker of cell death, was not increased until very high MCNB concentrations (≥3 mM) were used. Thus, MCNBs appear to be mild nephrotoxicants in vitro as well.

Studies on the effects of polychlorinated nitrobenzenes on renal function are limited to the work of Hong et al. who also determined the in vitro nephrotoxicity induced by the six DCNBs and two of the TCNBs [12]. The DCNBs were generally more potent nephrotoxicants than the MCNBs with only 3,4-dichloronitrobenzene (3,4-DCNB) reducing pyruvate-stimulated gluconeogenesis and increasing LDH release at a concentration as low as 0.5 mM. The two TCNBs tested (2,3,4- and 3,4,5-TCNB) were both potent nephrotoxicants and similar in their cytotoxic profile to 3,4-DCNB [12].

The role of biotransformation in chloronitrobenzene-induced nephrotoxicity is unknown. Chloronitrobenzenes are primarily metabolized by reduction of the nitro group to form the corresponding chloroaniline, followed by oxidation, acetylation, and conjugation reactions [16,17,18]. In addition, a chloro group in the para position to the nitro group can be displaced enzymatically by glutathione, followed by metabolism of the conjugate to a mercapturate metabolite [18,19]. The formation of mercapturate metabolites from a chloronitrobenzene is considered a biomarker of chloronitrobenzene exposure [20]. While chloroanilines are nephrotoxicants in vivo and in vitro [21,22,23,24,25], it is not clear if these metabolites contribute to chloronitrobenzene nephrotoxicity.

**Figure 1:**
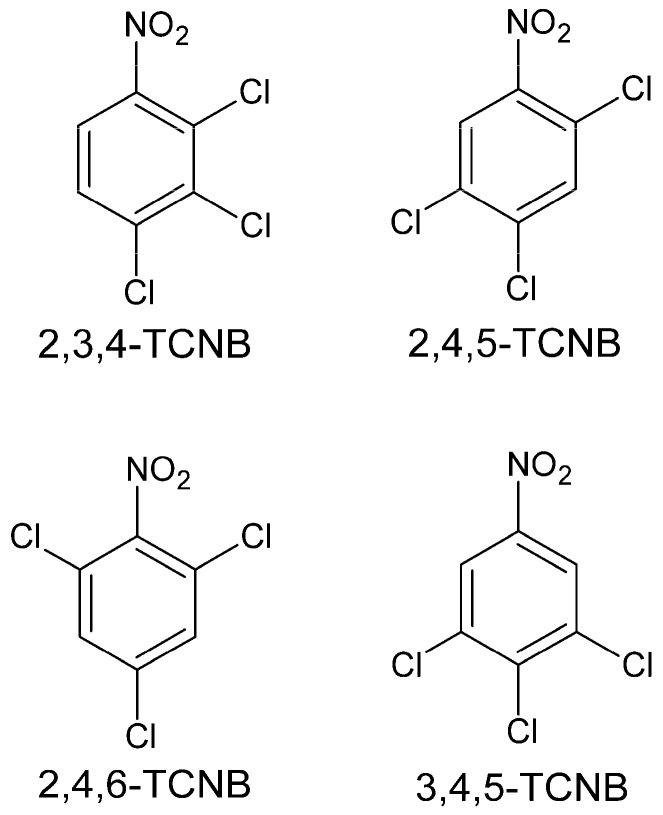
Structures of the four trichloronitrobenzenes (TCNBs) used in this study.

The purpose of this study was to extend earlier studies of TCNB in vitro nephrotoxicity by determining the nephrotoxic potential of four TCNB compounds (2,3,4-, 2,4,5-, 2,4,6-, and 3,4,5-TCNB; Figure 1) using isolated renal cortical cells (IRCC) as the animal model. The role of free radicals and/or biotransformation in TCNB in vitro nephrotoxicity was also examined. The IRCC model was chosen for study because many of our recent studies on chloroaniline nephrotoxicity have used this model [25]. The Fischer 344 rat was chosen because this rat strain responds to nephrotoxicants more like humans than other rat strains [26].

## 2. Results

### 2.1. Time and Concentration Cytotoxicity Studies

**Figure 2:**
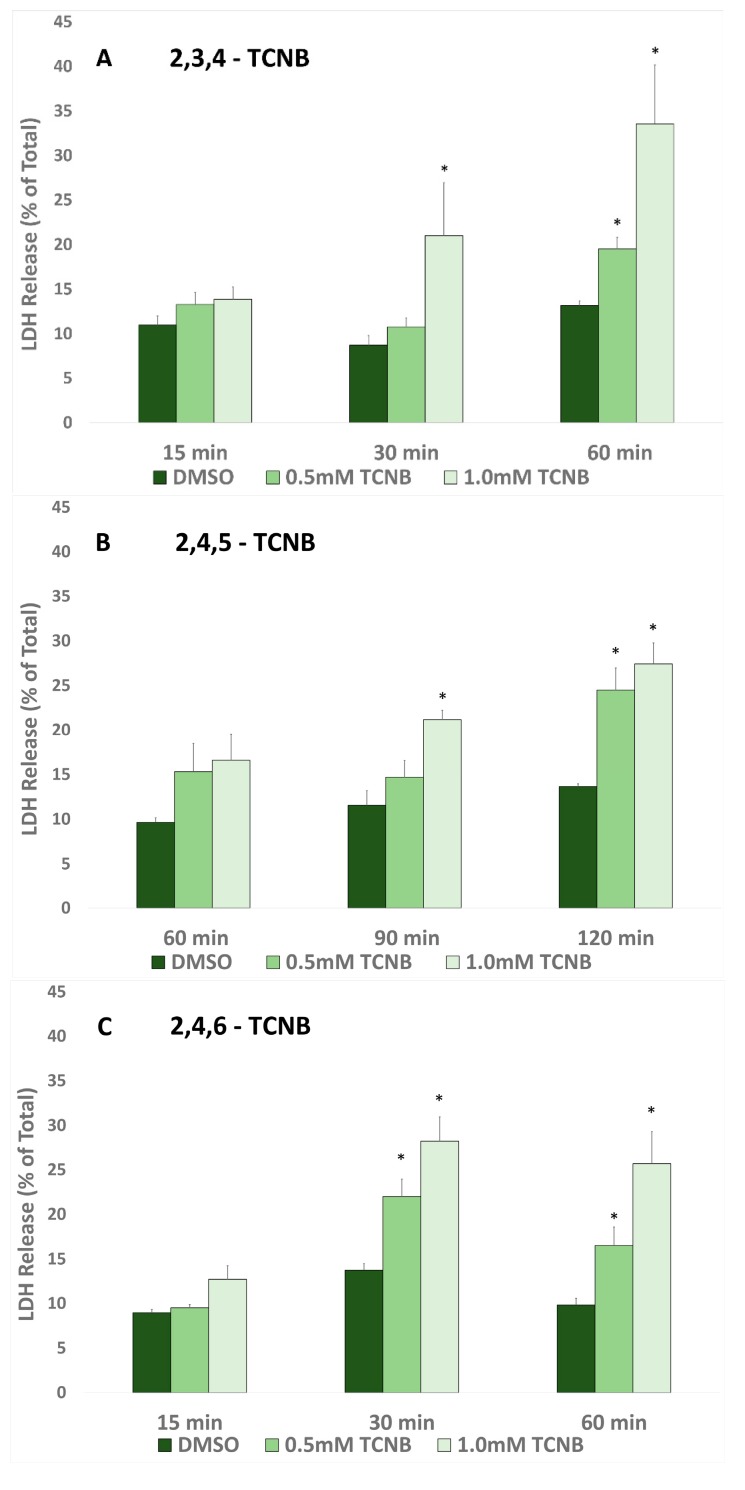

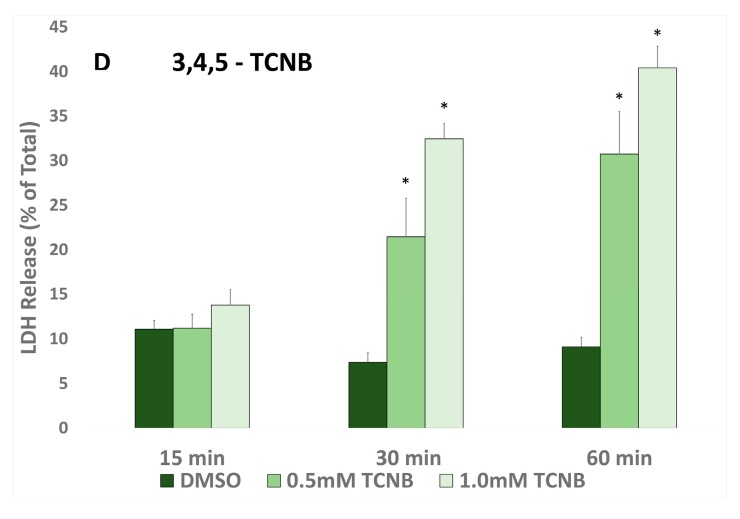
Cytotoxic effects of (**A**) 2,3,4-TCNB, (**B**) 2,4,5-TCNB, (**C**) 2,4,6-TCNB and (**D**) 3,4,5-TCNB in isolated renal cortical cells (IRCC). An asterisk (*) indicates significant difference from the appropriate dimethyl sulfoxide (DMSO) control group value, *p* < 0.05.

To determine the comparative nephrotoxic potential of the four TCNBs in IRCC, a time and concentration response study was performed starting at 15 min and 0.5 or 1.0 mM and adjusting the times tested based on the results (Figure 2). 

IRCC exposed to 2,3,4-TCNB (Figure 2A) exhibited cytotoxicity starting after 15 min (1.0 mM) and 60 min exposure (0.5 and 1.0 mM), but no cytotoxicity was observed at 15 min. 2,4,5-TCNB (Figure 2B) induced cytotoxicity at 90 min (0.5 mM) and 120 min (0.5 and 1.0 mM), but not at 60 min with either concentration. 2,4,6-TCNB (Figure 2C) cytotoxicity was observed at 30 and 60 min (0.5 and 1.0 mM) but not at 15 min with either concentration. 3,4,5-TCNB (Figure 2D) also induced cytotoxicity at 30 and 60 min (0.5 and 1.0 mM) but not at 15 min. Thus, the order of decreasing nephrotoxic potential of the four TCNBs tested was approximately 3,4,5- > 2,4,6- > 2,3,4- > 2,4,5-TCNB. Based on these findings, concentrations and incubation times were selected for evaluating the role of free radicals and biotransformation in the cytotoxicity induced by each of the four TCNBs, and 3,4,5-TCNB was selected for additional studies with more specific CYP inhibitors.

### 2.2. Effects of Antioxidants and Biotransformation Inhibitors on 2,3,4-Trichloronitrobenzene (2,3,4-TCNB) Cytotoxicity

Based on the results from the temporal and concentration studies, further studies with 2,3,4-TCNB to examine the role of free radicals and biotransformation in 2,3,4-TCNB cytotoxicity were conducted at 1.0 mM 2,3,4-TCNB and 60 min exposure. Pretreatment with any of the four antioxidants (α-tocopherol, ascorbate, glutathione, or *N*-acetyl-l-cysteine (NAC)) resulted in the attenuation of 2,3,4-TCNB cytotoxicity suggesting that free radicals were involved in the cytotoxic mechanism (Table 1). The 2,3,4-TCNB-induced cytotoxicity was also reduced by pretreatment with several of the biotransformation inhibitors including piperonyl butoxide and isoniazid (CYP inhibitors), methimazole and n-octylamine (FMO inhibitors), and indomethacin and mercaptosuccinate (peroxidase inhibitors). These results suggest that 2,3,4-TCNB cytotoxicity can involve multiple enzymes that can be involved in oxidation and/or reduction biotransformation reactions.

### 2.3. Effects of Antioxidants and Biotransformation Inhibitors on 2,4,5-TCNB Cytotoxicity

Based on the results from the temporal and concentration studies, further studies with 2,4,5-TCNB to examine the role of free radicals and biotransformation in 2,4,5-TCNB cytotoxicity were conducted at 1.0 mM 2,4,5-TCNB and 90 min exposure. As with 2,3,4-TCNB, pretreatment with any of the four antioxidants (α-tocopherol, ascorbate, glutathione, or NAC) resulted in attenuation of 2,4,5-TCNB cytotoxicity suggesting that free radicals were involved in the cytotoxic mechanism (Table 2). For the inhibition of biotransformation, 2,4,5-TCNB-induced cytotoxicity was only reduced by pretreatment with isoniazid (a CYP inhibitor). These results suggest that 2,4,5-TCNB cytotoxicity involves free radicals, but the role of biotransformation enzymes involved in oxidation and/or reduction biotransformation reactions in 2,4,5-TCNB cytotoxicity is less clear.

### 2.4. Effects of Antioxidants and Biotransformation Inhibitors on 2,4,6-TCNB Cytotoxicity

Based on the results from temporal and concentration studies, further studies with 2,4,6-TCNB to examine the role of free radicals and biotransformation in 2,4,6-TCNB cytotoxicity were conducted at 1.0 mM 2,4,6-TCNB and 90 min exposure. Pretreatment with ascorbate or glutathione resulted in attenuation of 2,4,6-TCNB cytotoxicity suggesting that free radicals were involved in the cytotoxic mechanism (Table 3). However, unlike 2,3,4- and 2,4,5-TCNB, cytotoxicity was not reduced by pretreatment with α-tocopherol or NAC. 

For the inhibition of biotransformation, 2,4,6-TCNB-induced cytotoxicity was only reduced by pretreatment with metyrapone (a CYP inhibitor), indomethacin (a peroxidase inhibitor), and methimazole (a FMO inhibitor). These results suggest that 2,4,6-TCNB cytotoxicity involves free radicals, and biotransformation reactions contribute to 2,4,6-TCNB cytotoxicity.

### 2.5. Effects of Antioxidants and Biotransformation Inhibitors on 3,4,5-TCNB Cytotoxicity

**Figure 3:**
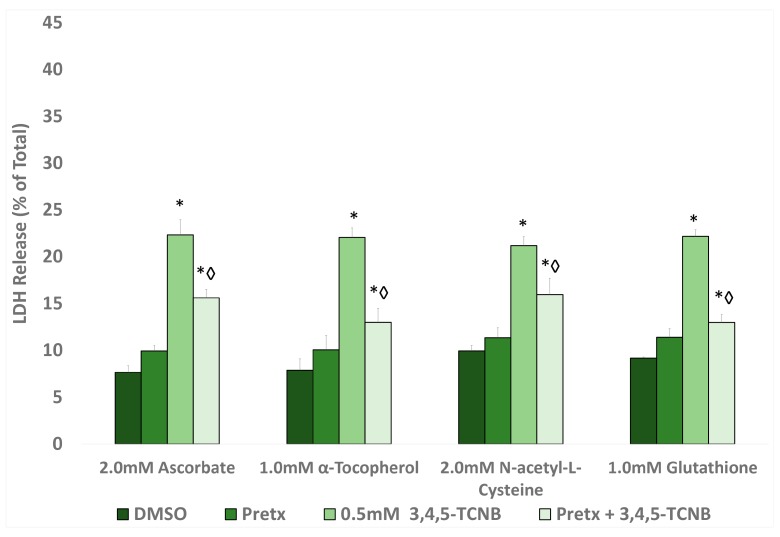
Effect of antioxidant pretreatment on 3,4,5-TCNB 0.5 mM cytotoxicity after a 30 min 3,4,5-TCNB incubation period. An * indicates significant difference from the DMSO control value, *p* < 0.05. A diamond indicates significant difference from the 3,4,5-TCNB group only value, *p* < 0.05. *n* = 4.

**Figure 4:**
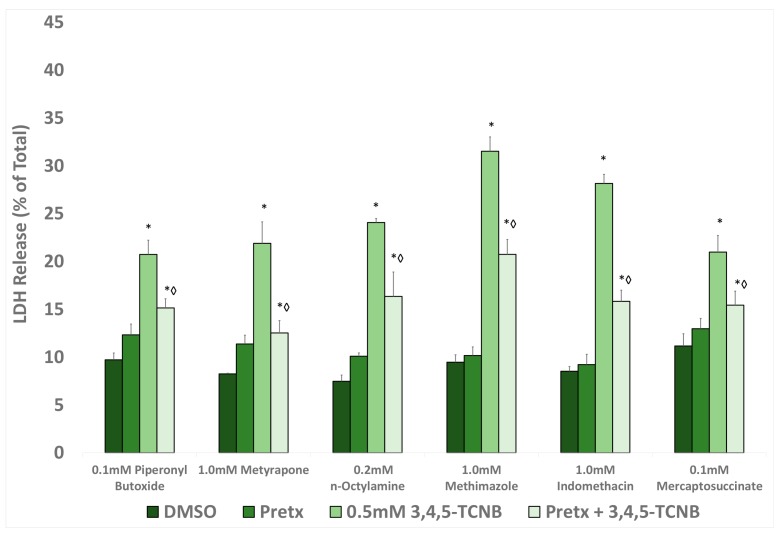
Effects of pretreatment with a cytochrome P450 (CYP) inhibitor (piperonyl butoxide, metyrapone), flavin monooxygenase (FMO) inhibitor (n-octylamine, methimiazole), or peroxidase inhibitor (indomethacin, mercaptosuccinate) on 3,4,5-TCNB 0.5 mM cytotoxicity after a 30 min 3,4,5-TCNB incubation period. An * indicates significant difference from the DMSO control value, *p* < 0.05. A diamond indicates significant difference from the 3,4,5-TCNB group only value, *p* < 0.05. *n* = 4.

Based on the results from the temporal and concentration studies, further studies with 3,4,5-TCNB to examine the role of free radicals and biotransformation in 3,4,5-TCNB cytotoxicity were conducted at 0.5 mM 3,4,5-TCNB and 30 min exposure. Pretreatment with any of the four antioxidants (α-tocopherol, ascorbate, glutathione, or NAC) resulted in attenuation of 3,4,5-TCNB cytotoxicity, suggesting that free radicals were involved in the cytotoxic mechanism (Figure 3). The 3,4,5-TCNB-induced cytotoxicity was also reduced by pretreatment with a biotransformation inhibitor including piperonyl butoxide and metyrapone (CYP inhibitors), methimazole and n-octylamine (FMO inhibitors), and indomethacin and mercaptosuccinate (peroxidase inhibitors) (Figure 4).

**Figure 5:**
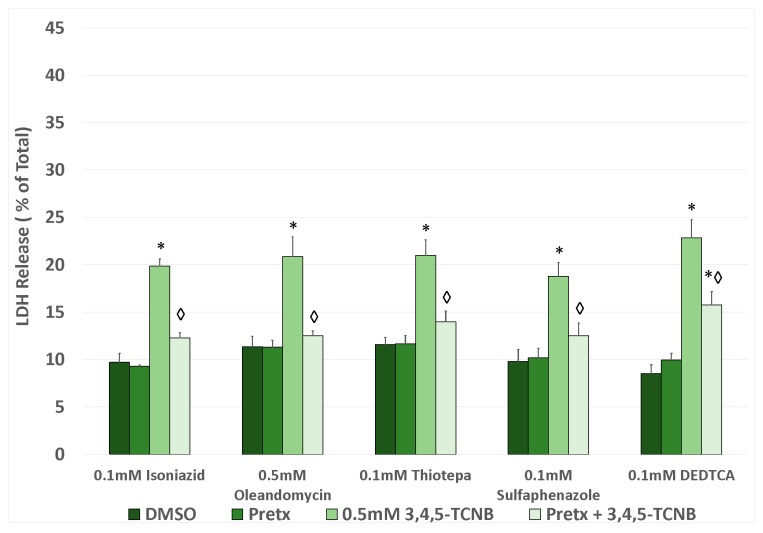
Effects of pretreatment with a CYP specific inhibitor on 3,4,5-TCNB 0.5 mM cytotoxicity after a 30 min 3,4,5-TCNB incubation period. An * indicates significant difference from the DMSO control value, *p* < 0.05. A diamond indicates significant difference from the 3,4,5-TCNB group only value, *p* < 0.05. *n* = 4.

To explore the specificity of CYP isozymes for attenuating 3,4,5-TCNB cytotoxicity, the pretreatment of IRCC with several selective CYP inhibitors was evaluated to determine the ability of any of these specific inhibitors to attenuate 3,4,5-TCNB cytotoxicity. These inhibitors included isoniazid (CYP2E1), oleandomycin (CYP3A1/2), thio-tepa (CYP2B1/2), sulfaphenazole (CYP2C), and diethyldithiocarbamate (CYP2C>2E1). All of the inhibitors offered at least some attenuation of 3,4,5-TCNB cytotoxicity (Figure 5). These results suggest that 3,4,5-TCNB cytotoxicity can involve bioactivation by multiple enzymes that can be involved in oxidation and/or reduction biotransformation reactions.

To determine the effect of 3,4,5-TCNB 1.0 mM on adenosine triphosphate (ATP) and adenosine diphosphate (ADP) levels at toxic and nontoxic time points, cells were treated with 3,4,5-TCNB for 15 or 30 min. The results demonstrate that 3,4,5-TCNB at a toxic concentration reduces ATP levels at 15 min, which is prior to cell death (Table 4). This result suggests that mitochondria could be a target for the ultimate toxic TCNB metabolite(s).

**Figure 6:**
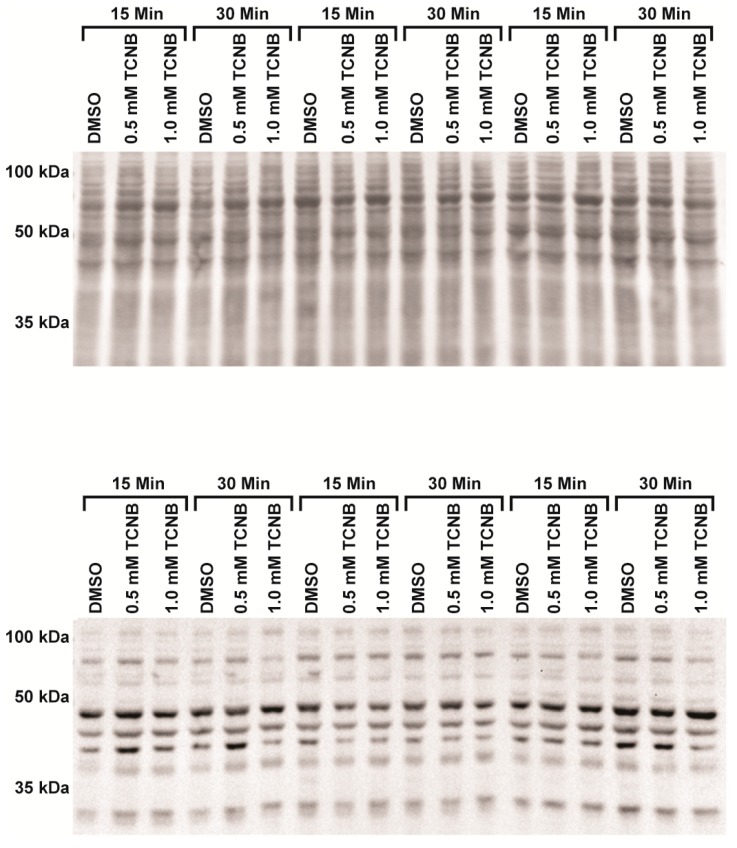
Effect of 3,4,5-TCNB treatment on 4-HNE adduction in IRCC from three different animals. The top panel represents the total protein, while the bottom panel represents the 4-HNE adducted proteins. *n* = 3 separate experiments.

To determine if oxidative stress plays a role in 3,4,5-TCNB nephrotoxicity, experiments were conducted to determine the temporal aspects of free radical damage as evidenced by protein carbonylation (OxyBlot) and 4-hydroxynonenal (4-HNE) adduction. As can be seen from Figure 6, 4-HNE levels were not increased by 0.5 or 1.0 mM 3,4,5-TCNB at 15 or 30 min.

**Figure 7:**
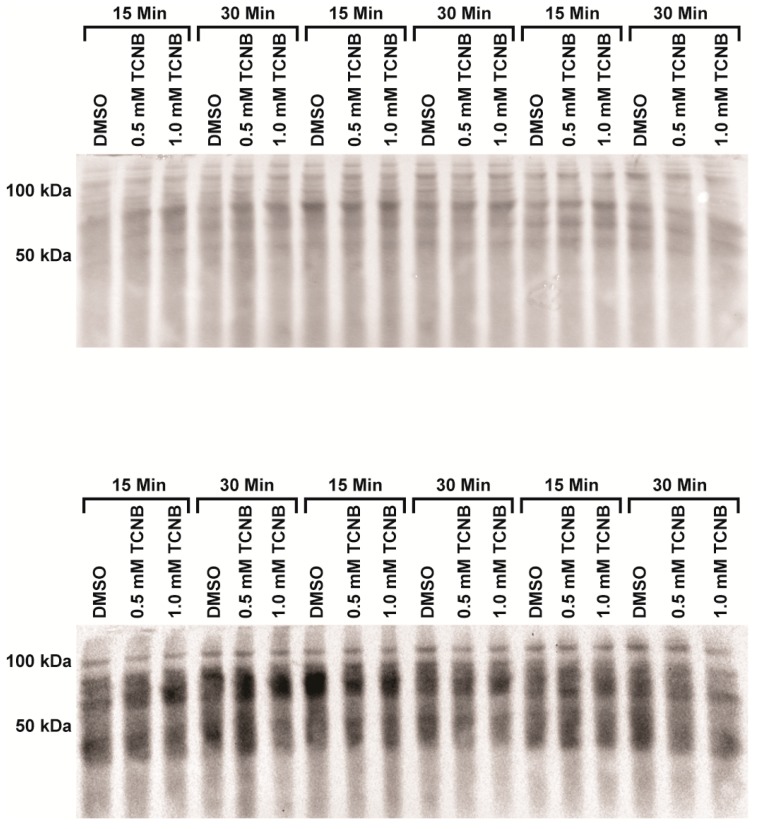
Effect of 3,4,5-TCNB treatment on protein carbonylation in IRCC from three different animals using OxyBlot. The top panel represents the total protein, while the bottom panel represents the carbonylated proteins. *n* = 3 separate experiments.

Protein carbonylation was also not increased by 3,4,5-TCNB treatment at 0.5 or 1.0 mM and slightly decreased at 30 min in the 1.0 mM 3,4,5-TCNB group (*p* = 0.017), Figure 7. Collectively, these results suggest that lipid peroxidation is not the primary mechanism of cell killing and that other mechanisms are responsible for 3,4,5-TCNB cytotoxicity in IRCC.

## 3. Discussion

There is little toxicological information available on the potential harmful effects of the TCNBs in mammals. Hong et al. [24] demonstrated that two TCNBs (2,3,4- and 3,4,5-TCNB) induced in vitro nephrotoxicity (increased LDH release and decreased pyruvate-stimulated gluconeogenesis) at concentrations as low as 0.5 mM after 120 min incubations in renal cortical slices from male Fischer 344 rats. In the present study using IRCC from male Fischer 344 rats, it was determined that TCNBs are also directly toxic to IRCC, and the order of decreasing nephrotoxic potential of the four TCNBs tested was approximately 3,4,5- > 2,4,6- > 2,3,4- > 2,4,5-TCNB. Cytotoxicity was evident at 0.5 mM and as early as 30 min with both 3,4,5- and 2,4,6- TCNB in IRCC, suggesting that IRCC might be a more sensitive model for measuring the nephrotoxic effects of the TCNBs than renal cortical slices.

The ability of many of the inhibitors used in this study to attenuate TCNB cytotoxicity suggests that toxic metabolites of TCNBs contribute to TCNB in vitro nephrotoxicity. The biotransformation of chloronitrobenzenes has been studied in a number of species, with most of the studies to date having focused on the metabolism of MCNBs in rats [27,28,29], rabbits [30], or humans [17,18,20]. In general, a MCNB undergoes three types of initial biotransformation reactions; (1) oxidation of the aromatic ring in a ring position ortho to a chloro group and meta to the nitro group, (2) displacement of a chloro group ortho or para to the nitro group by glutathione conjugation and (3) reduction of the nitro group to form a chloroaniline. The glutathione conjugates are further metabolized to mercapturate metabolites, which can be used as biomarkers of exposure to a chloronitrobenzene [17,20]. Reduction of the nitro group to form an aniline appears to be a major metabolic pathway in all species studied. The chloroanilines can be further metabolized to aminophenols, acetanilides, and oxanilic acids, plus glucuronide and sulfate conjugates [18,27,29]. At least with 4-chloronitrobenzene, acetanilide and aminophenol formation was encountered less frequently in humans than in rats [17].

**Figure 8:**
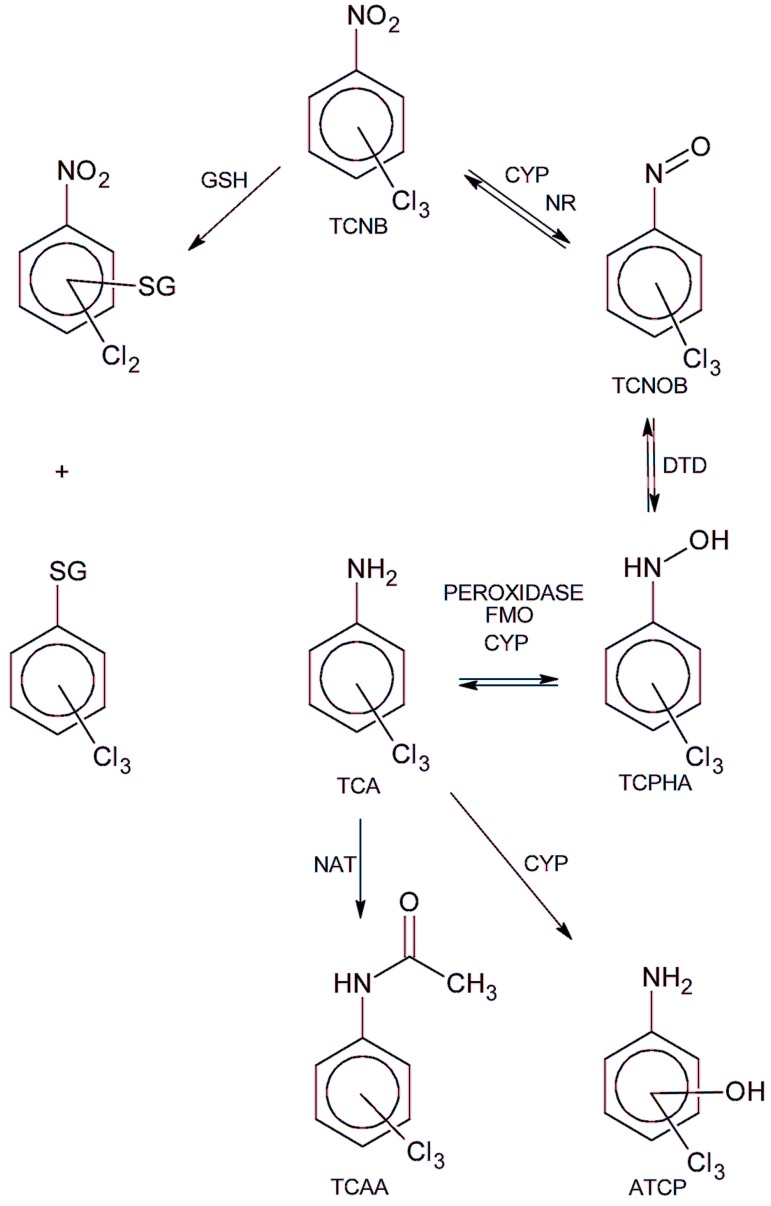
Putative biotransformation pathway for the TCNBs. ATCP = aminotrichlorophenol, CYP = cytochrome P450, DTD = DT-diaphorase, FMO = Flavin monooxygenase, GSH = glutathione, NAT = *N*-acetyltransferase, NR = nitroreductase, TCA = trichloroanilines, TCAA = trichloroacetanilide, TCNOP = trichloronitrosobenzene, TCPHA = trichlorophenylhydroxylamine.

In the one study of TCNB metabolism, Betts et al. examined the biotransformation of the six TCNBs in rabbits following oral administration (0.2–0.8 g/kg) of a TCNB by determining the identity of urinary metabolites at 24 h intervals for three days [16]. In general, the metabolic pathways determined for the TCNBs in rabbit were very similar to the pathways identified for MCNBs (nitro reduction, glutathione conjugation of the TCNB, and aminochlorophenol formation), with the addition of hexachloroazoxybenzenes being formed as minor metabolites from 2,4,5- and 3,4,5-TCNB. Although TCNB metabolism by the kidney in rats has not been determined, a putative metabolic pathway for the TCNBs in rat kidney could be proposed (Figure 8) based on the results of these previous studies.

It is unlikely that glutathione conjugation of a TCNB alone leads to toxic metabolites. Glutathione conjugation is normally a detoxifying mechanism that reduces the ability of electrophiles to react with cellular macromolecules. In cases where glutathione conjugates are nephrotoxiciants, the conjugates are produced extrarenally, and the glutathione or resultant mercapturate conjugate provides a means for renal accumulation and/or bioactivation [31]. In this study, the TCNB would be expected to enter the IRCC via passive diffusion prior to metabolism and not require a glutathione derived conjugate to promote uptake. Thus, it is unlikely that the ultimate toxic metabolite results from glutathione conjugation.

*N*-Acetylation of an aniline metabolite to form a chloroacetanilide is also not a likely bioactivation mechanism. *N*-Acetylation is catalyzed in kidney cells by cytosolic *N*-acetyltransferase enzymes, and it is very unlikely that the pretreatments used in this study that attenuated TCNB cytotoxicity would significantly alter this metabolism reaction. In addition, chloroacetanilides have greatly reduced nephrotoxic potential in vivo when compared to the parent chloroanilines or aminochlorophenol metabolites [32,33]. Thus, it is unlikely that acetylation of any aniline derived metabolites would be a mechanism for bioactivation.

Reduction of chloronitrobenzenes to chloroanilines is a common biotransformation pathway for the MCNBs and TCNBs [16,20,27]. Reduction of the nitro group in chloronitrobenzenes is catalyzed by microsomal enzymes (CYPs and nitroreductases) and is inhibited by cytochrome P450 (CYP) inhibitors [27]. As the nitro group is reduced, chloronitrosobenzenes and then chlorophenylhydroxylamines are formed before the final reduction to the chloroaniline metabolite (Figure 6). During the reduction, electrons can be transferred to oxygen to form the superoxide anion radical, which could lead to oxidative damage and cell death. The chloroaniline metabolites can also be oxidized back to the corresponding chloronitrobenzene through a reverse set of metabolic steps. Oxidation of the chloroaniline metabolite to an *N*-hydroxyl metabolite (Figure 6.) can be catalyzed by several enzyme systems including CYPs, flavin monooxygenases (FMOs), prostaglandin H synthase, and other peroxidases and contribute to chloroaniline toxicity [34,35,36,37,38,39,40,41]. Further oxidation of the chlorophenylhydroxylamine metabolites can occur via auto-oxidation. The result is that both chloroanilines and chloronitrobenzenes have the potential to set up a redox situation involving the production of chloronitrosobenzenes and chlorophenylhydroxylamines that could produce free radicals that could lead to potential oxidative damage and cellular toxicity. In erythrocytes, this redox cycling leads to methemoglobin formation via oxidation of the ferrous iron in hemoglobin as the various metabolites are reduced, and oxidative and reactive metabolites can eventually lead to the destruction of the erythrocytes and anemia [9,42,43]. In addition, chloronitrosobenzenes are reactive compounds that can bind to cellular macromolecules and contribute to the cellular toxicity of these compounds [44,45,46,47]. Thus, the reversible metabolism of the TCNBs to trichloroanilines is a potential pathway for generating the nephrotoxic metabolites responsible for TCNB cytotoxicity observed in this study.

The results of this study demonstrated that cytotoxicity induced by 2,3,4-, 2,4,6-, and 3,4,5-TCNB was inhibited by pretreatment with at least one CYP inhibitor, FMO inhibitors, and peroxidase inhibitors, suggesting that the nitro reduction-amino oxidation pathway was contributing to the production of nephrotoxic metabolites. The placement of the three chloro groups on the TCNB appears to affect the ability of the TCNB to serve as a substrate for particular oxidation/reduction metabolizing enzymes, although no clear correlation pattern of substitution emerged. The nitro and resulting amino group in the aniline metabolite of 3,4,5-TCNB are the least sterically hindered and most accessible to metabolizing enzymes, and 3,4,5-TCNB is the most nephrotoxic of the four compound studies. However, although 2,4,6-TCNB was close to 3,4,5-TCNB in cytotoxic potential at 30 min (Figure 2), two of the three chloro groups are ortho to the nitro group and provide the most steric hindrance for metabolizing the nitro group. Given the contributions of multiple enzyme systems contributing to TCNB metabolism, it is possible that the chloro groups, depending on ring location, direct the TCNBs and/or their metabolites to only certain metabolizing enzymes. It also appears that 3,4,5-TCNB may be metabolized by multiple CYPs to the ultimate toxic metabolite(s), as all of the specific CYP inhibitors used in this study offered some protection, or that multiple metabolic steps, catalyzed by different CYPs are required to produce the ultimate toxicant. However, more work needs to be done to establish the effect that the chloro group position on the aromatic ring has on the biotransformation and nephrotoxic potential of TCNBs.

The contributions of aminochlorophenol metabolites to TCNB nephrotoxicity must also be considered. Aminochlorophenols are known nephrotoxicants in vivo and in vitro in Fischer 344 rat models [48,49,50,51], and these metabolites are known to be produced in rats [18], rabbits [16], and humans [17]. However, studies with 3,5-dichloroaniline and 3,4,5-trichloroaniline, the most potent nephrotoxicants among the di- and trichloroanilines isomers, in Fischer 344 rats have shown that in vitro, *N*-oxidation pathways are more important than the formation of aminophenol metabolites for inducing nephrotoxicity [25,52]. Thus, while TCNBs may be metabolized to trichloroanilines in the rat kidney, *N*-oxidation is the most likely pathway leading to nephrotoxic metabolites. Nonetheless, the contribution of aminophenol metabolites to TCNB nephrotoxicity needs to be studied further.

The ability of the four antioxidants (α-tocopherol, ascorbate, glutathione, and NAC) to attenuate the cytotoxicity of the four TCNBs provides insight into the role of free radicals in the toxicity induced by these compounds. Ascorbate is a water soluble radical scavenger that can quench radicals in solution. α-Tocopherol is a fat soluble antioxidant that primarily works by helping neutralize lipid peroxide formation and the generation of reactive oxygen species from oxidized lipids. NAC serves as an antioxidant primarily following the conversion to glutathione, but can, to a lesser extent, form disulfides (*N*,*N*-diacetylcystine) in the presence of free radicals to scavenge those radicals. Glutathione can neutralize free radicals (via glutathione peroxidase) or electrophiles (via glutathione S-transferase). Occasionally, NAC is not an effective protectant, when glutathione is protective. This observation could be due to the time required to effectively convert NAC to glutathione and/or to the fact that the cytotoxic chemical species is a reactive metabolite rather than a free radical and more easily neutralized by enzymatic conjugation with glutathione.

All four antioxidants attenuated the cytotoxicity caused by 2,3,4-, 2,4,5-, and 3,4,5-TCNB at the concentrations and times studied. Free radicals may be produced during the reduction of the nitro group or during the oxidation of trichloroaniline metabolites [53], or may be the result of redox cycling of aminochlorophenol or chlorophenylhydroxylamine/chloronitrosobenzene metabolites to produce reactive oxygen species (ROS) and oxidative damage [54,55]. While some degree of oxidative stress may be induced by these compounds, the current work suggests that, at least for 3,4,5-TCNB, oxidative stress-induced lipid peroxidation is not the mechanism of cytotoxicity. The absence of an increase in 4-HNE or protein carbonylation, even at a time when 3,4,5-TCNB induced cytotoxicity, indicates that cells are dying by other mechanisms and antioxidants are attenuating TCNB cytotoxicity via mechanisms not involving quenching ROS or ROS-derived intermediates. Similarly, the finding that ascorbate but not α-tocopherol is protective against 2,4,6-TCNB could suggest that radical TCNB metabolites are formed but these metabolites are not leading to lipid peroxidation. A similar observation was noted for 3,5-dichloroaniline [52], suggesting that chloroanilines and chloronitrobenzenes may share a common metabolic pathway, such as the generation of chloronitrosobenzenes, to induce nephrotoxicity. Additional work is needed to clarify if either or both of these potential pathways of free radical generation can explain the mechanism of protection by antioxidants on TCNB cytotoxicity and provide insights into the cellular targets and mechanism of TCNB nephrotoxicity.

## 4. Materials and Methods

### 4.1. Experimental Animals

Male Fischer 344 rats (200–260 g) obtained from Hilltop Lab Animals (Scottdale PA) were used for all experiments. All animals were maintained under a controlled environment consisting of a controlled light cycle (on 12 h, off 12 h), temperature (21–23 °C), and humidity (40–55%). Rats were housed in standard plastic cages (two rats per cage) prior to use. Rats were allowed to acclimate to the animal facilities for at least one week before use in experiments. Purina Rat Chow and water were available ad libitum. The Marshall University Institutional Care and Use Committee approved all animal use protocols. Studies were performed at an AAALAC (Association for the Assessment and Accreditation of Laboratory Animal Care International) accredited facility and all animal care was in accordance with the American Association for Laboratory Animal Science (AALAS) Policy on the Human Care and Use of Laboratory Animals (http://www.aalas.org).

### 4.2. Chemicals

Chemicals used in this study were of the highest purity available, with 2,3,4- and 3,4,5-TCNB purchased from Sigma Aldrich (St. Louis, MO, USA) and 2,4,5- and 2,4,6-TCNB purchased from Tokyo Chemical Industry Co., Ltd. (Tokyo, Japan).

### 4.3. Isolated Renal Cortical Cell (IRCC) Preparation and Treatment

Untreated rats were anesthetized with pentobarbital (75 mg/kg, i.p.) and isolated renal cortical cells (IRCC) were obtained by using the collagenase perfusion method [56]. Initial cell viability was determined by measuring trypan blue (2% *w*/*v*) exclusion and lactate dehydrogenase (LDH) release. IRCC were counted and re-suspended in Krebs-Henseleit buffer, pH 7.4, containing 25 mM Hepes and 2% (*w*/*v*) bovine serum albumin at a concentration of ~4.0 million cells/mL. IRCC (3 mL) were then added to a polycarbonate Erlenmeyer flask (25 mL) for 5 min in a shaking water bath incubator (37 °C, 60 cycles/min) under a 95% oxygen/5% carbon dioxide atmosphere. IRCC were then exposed to various concentrations of a TCNB (0, 0.5, or 1.0 mM) for 15, 30, 60, 90, or 120 min. Following the incubation period, flasks were removed and placed on ice. Samples (0.5 mL) were taken for LDH release assays. Briefly, the samples were centrifuged (3000× *g*, 3 min), the was supernatant decanted and saved, and the pellet was disrupted with 1 mL of 10% Triton X-100 solution to release cellular LDH activity. LDH activity was then determined in each fraction (supernatant and pellet) as previously described using a kinetic assay based on the amount of NADH produced from NAD [57]. The LDH released was expressed as % of total (supernatant plus pellet).

In separate experiments, IRCC were pretreated with either an antioxidant or a biotransformation enzyme system inhibitor before exposure to a toxic concentration of a TCNB followed by incubation at times where toxicity was observed. Concentrations used in these experiments were 0.5 mM (3,4,5-TCNB) or 1.0 mM (2,3,4-, 2,4,5- and 2,4,6-TCNB). Incubation times with a TCNB added were 30 min (3,4,5-TCNB), 60 min (2,3,4-TCNB), or 90 min (2,4,5- and 2,4,6-TCNB). The pretreatment times and concentrations for all of the pretreatments are shown below in Table 5.

### 4.4. Adenine Nucleotides

Upon completion of the incubation of IRCC with 3,4,5-TCNB or vehicle as described above, a 500 µL aliquot of cell suspension was centrifuged for 10 min at 2000× *g* at 4 °C. The pellet was rinsed with 1 mL Krebs buffer, centrifuged, and the pellet treated with 150 µL lysis buffer (Cell Signaling) followed by the addition of 200 µL of 3 N perchloric acid. The suspension was vortexed and placed on ice for 5 min to precipitate all protein followed by centrifugation for 10 min at 2000× *g* at 4 °C. A 175 µL aliquot of the supernatant was adjusted to pH 6.5 with 3 N KOH [68]. The supernatant was filtered (0.45 mm syringe filters) and 10 µL were injected onto a Waters Alliance e2695 HPLC system. The column was a Waters xBridge C18 5 micron particle size column with the dimensions of 100 × 4.6 mm inner diameter. The UV variable wavelength detector was set at 254 nm. The mobile phase was a gradient of 100% 0.1 M potassium phosphate buffer from 0–3 min followed by a 1 min gradient to 90:10 phosphate buffer:methanol followed by a 4 min gradient to 100% phosphate buffer at a constant flow of 0.8 mL/min. ATP and ADP were used to generate a linear standard curve with a representative regression analysis of *r*^2^ = 0.9974 and 0.9992, respectively.

### 4.5. Western Blot, Protein Carbonylation (OxyBlot™), and 4-HNE

Protein carbonylation was detected using OxyBlot™ Protein Oxidation (Millipore S7150; Temecula, CA, USA). Samples were prepared according to the manufacturer’s protocol and an equal amount of protein (25 µg) and volume were loaded in each lane of a 12.5% polyacrylamide gel and transferred to a nitrocellulose membrane (Whatman; 10-439-196; Dassel, Germany). The transfer was confirmed using a Memcode reversible protein stain kit (Pierce Biotechnology, Fisher Scientific) and the protein was used to normalize each lane. Membranes were incubated overnight at 4 °C with primary antibody (1:150) for OxyBlot. Goat anti-rabbit horse radish peroxidase (HRP, 1:300) was the secondary antibody. Protein with 4-hydroxynonenal (4-HNE) reduced Micahel adducts were prepared as described previously [69] with the exception that 45 µg of protein was used for the gel. The membranes were developed using Amersham^TM^ ECLTM Western Blotting Reagents (GE Healthcare; Buckinghamshire, UK) and the results were viewed and analyzed using a Bio-Rad Chemidoc system with BioRad Chemidoc densitometry software (version 4.0.1, Catalog No. 170-9690, BioRad, Hercules, CA, USA).

### 4.6. Statistics

Data are shown as the mean ± S.E.M. with an *n* = 4. Data were analyzed by one-way analysis of variance followed by a Student-Newman-Keuls Test. Differences were considered to be significant at *p* < 0.05, α = 0.5.

## 5. Conclusions

The results of the temporal and concentration studies determined that the in vitro decreasing order of nephrotoxicity of the four TCNBs was approximately: 3,4,5- > 2,4,6- > 2,3,4- > 2,4,5-TCNB based on time of onset of cytotoxicity, lowest concentration required for cytotoxicity, and the magnitude of LDH release. Free radicals appear to play a role in the cytotoxicity induced by the TCNBs, and may be produced during biotransformation of the TCNBs. The production of free radicals leading to oxidative stress and lipid peroxidation does not appear to be the primary mechanism of cytotoxicity. However, ATP depletion occurs prior to LDH release for 3,4,5-TCNB, which could indicate that mitochondria are targets for TCNB or its metabolites. It was also determined that biotransformation to toxic metabolites is important for TCNB-induced nephrotoxicity and that pathways involving reduction of the nitro group and/or oxidation of the resulting trichloroanilines are the most likely to be producing the nephrotoxicant metabolites. Positioning of the three chloro groups also appears to dictate which bioactivating enzyme systems contribute to TCNB metabolism. However, the ultimate nephrotoxicant species from TCNBs remains to be determined with certainty.

## Figures and Tables

**Table 1:** Effect of antioxidants and metabolism inhibitors on 2,3,4-trichloronitrobenzene (2,3,4-TCNB) cytotoxicity ^a^.

Pretreatment ^b^	Vehicle	PreTx	2,3,4-TCNB	PreTx + TCNB
Ascorbate	12 ± 1	12 ± 1	20 ± 2 ^c^	14 ± 1 ^d^
α–Tocopherol	11 ± 1	11± 1	23 ± 2 ^c^	12 ± 2 ^d^
Glutathione	12 ± 1	13 ± 1	20 ± 1 ^c^	14 ± 1 ^d^
NAC	9 ± 2	13± 1	23 ± 2 ^c^	16 ± 1 ^c,d^
PiBx	8 ±1	10 ± 1	21 ± 1 ^c^	12 ± 1 ^c,d^
Metyrapone	11 ± 1	12 ± 1	19 ± 1 ^c^	17 ± 1 ^c^
Isoniazid	10 ± 1	11 ± 1	23 ± 2 ^c^	19 ± 1 ^c,d^
n-Octylamine	10 ± 1	16 ± 1 ^c^	28 ± 2 ^c^	19 ± 1 ^c,d^
Methimazole	8 ± 1	9 ± 1	21 ± 1 ^c^	11 ± 1 ^d^
Indomethacin	10 ± 1	13 ± 1	22 ± 1 ^c^	16 ± 2 ^c,d^
Mercaptosuccinate	9 ± 2	10 ± 1	18 ± 1 ^c^	10 ± 1 ^d^

^a^ Incubations were carried out for 60 min with 1.0 mM 2,3,4-TCNB, *n* = 4. Values are % of lactate dehydrogenase release ± S.E. Incubation times and inhibitor concentrations are provided in Materials and Methods. ^b^ NAC = *N*-acetyl-l-cysteine; PiBx = piperonyl butoxide; PreTx = pretreatment. ^c^ Significantly different from the vehicle control value, *p* < 0.05. ^d^ Significantly different from the TCNB only value, *p* < 0.05.

**Table 2:** Effect of antioxidants and metabolism inhibitors on 2,4,5-TCNB cytotoxicity ^a^.

Pretreatment ^b^	Vehicle	PreTx	2,3,4-TCNB	PreTx + TCNB
Ascorbate	11 ± 1	12 ± 1	22 ± 1 ^c^	16 ± 2 ^d^
α–Tocopherol	12 ± 1	13 ± 1	22 ± 1 ^c^	14 ± 3 ^d^
Glutathione	14 ± 1	15 ± 1	26 ± 3 ^c^	17 ± 2 ^d^
NAC	14 ± 1	15 ± 1	26 ± 2 ^c^	17 ± 1 ^d^
PiBx	9 ± 1	11 ± 2	27 ±2 ^c^	24 ± 3 ^c^
Metyrapone	9 ± 1	16 ± 1 ^c^	26 ± 2 ^c^	28 ± 2 ^c^
Isoniazid	9 ± 1	10 ± 1	28 ± 2 ^c^	34 ± 1 ^c,d^
n-Octylamine	9 ± 1	11 ± 2	28 ± 2 ^c^	32 ± 2 ^c^
Methimazole	12 ± 2	14 ± 2	28 ± 1 ^c^	23 ± 2 ^c^
Indomethacin	13 ± 1	16 ± 2	26 ± 1 ^c^	21 ± 2 ^c^
Mercaptosuccinate	13 ± 1	17 ± 1 ^c^	26 ± 1 ^c^	24 ± 1 ^c^

^a^ Incubations were carried out for 90 min with 1.0 mM 2,4,5-TCNB, *n* = 4. Values are % of lactate dehydrogenase release ± S.E. Incubation times and inhibitor concentrations are provided in Materials and Methods. ^b^ NAC = *N*-acetyl-l-cysteine; PiBx = piperonyl butoxide; PreTx = pretreatment. ^c^ Significantly different from the vehicle control value, *p* < 0.05. ^d^ Significantly different from the TCNB only value, *p* < 0.05.

**Table 3:** Effect of antioxidants and metabolism inhibitors on 2,4,6-TCNB cytotoxicity ^a^.

Pretreatment ^b^	Vehicle	PreTx	2,4,6-TCNB	PreTx + TCNB
Ascorbate	12 ± 1	12 ± 1	24 ± 1 ^c^	17 ± 2 ^c,d^
α–Tocopherol	13 ± 1	14 ± 1	30 ± 2 ^c^	25 ± 2 ^c^
Glutathione	12 ± 1	15 ± 1	30 ± 1 ^c^	20 ± 1 ^c,d^
NAC	13 ± 1	13 ± 1	28 ± 1 ^c^	23 ± 2 ^c^
PiBx	13 ± 1	13 ± 1	26 ± 2 ^c^	22 ± 1 ^c^
Metyrapone	11± 1	15 ± 1	22 ± 1 ^c^	27 ± 2 ^c^
Isoniazid	11 ± 1	10 ± 1	26 ± 3 ^c^	28 ± 2 ^c^
n-Octylamine	11 ± 1	11 ± 1	27 ± 3 ^c^	29 ± 3 ^c^
Methimazole	14 ± 1	17 ± 1	29 ± 2 ^c^	31 ± 2 ^c^
Indomethacin	11 ± 1	16 ± 1 ^c^	28 ± 2 ^c^	21 ± 1 ^c,d^
Mercaptosuccinate	14 ± 1	16 ± 1	28 ± 2 ^c^	31 ± 1 ^c^

^a^ Incubations were carried out for 90 min with 1.0 mM 2,4,6-TCNB, *n* = 4. Values are % of lactate dehydrogenase release ± S.E. Incubation times and inhibitor concentrations are provided in Materials and Methods. ^b^ NAC = *N*-acetyl-l-cysteine; PiBx = piperonyl butoxide; PreTx = pretreatment. ^c^ Significantly different from vehicle control value, *p* < 0.05. ^d^ Significantly different from TCNB only value, *p* < 0.05.

**Table 4:** Effect of 3,4,5-TCNB on ATP and ADP ^a^.

Treatment	Time (min)	ATP nmol/4 Million Cells	ADP nmol/4 Million Cells
DMSO	15	13.47 ± 1.65	14.21 ± 1.26
DMSO	30	12.74 ± 1.87	15.28 ± 1.23
3,4,5-TCNB 1.0 mM	15	7.93 ± 1.37 ^b^	13.74 ± 1.51
3,4,5-TCNB 1.0 mM	30	6.51 ± 0.98 ^b^	12.62 ± 1.63

^a^ Values are means ± S.E. for *n* = 6 experiments. ^b^ Significantly different from the DMSO value, *p* < 0.05.

**Table 5:** List of pretreatment antioxidants and enzyme inhibitors with primary targeted enzyme systems.

Pretreatment (PreTx) Compound	PreTx Conc.	PreTx Time (min)	Mechanism or Enzyme Inhibited	Reference
*N*-acetyl-l-cysteine (NAC)	2.0 mM	30	Antioxidant	[58]
α-Tocopherol	1.0 mM	5	Antioxidant	[59]
Glutathione	1.0 mM	30	Antioxidant	[60]
Ascorbate	2.0 mM	5	Antioxidant	[61]
Pyruvate	1.0 mM	15	Antioxidant	[55,62]
Methimazole	1.0 mM	30	FMO	[63]
*N*-Octylamine	2.0 mM	5	FMO	[63]
Indomethacin	1.0 mM	15	Cyclooxygenase	[60]
Mercaptosuccinate	0.1 mM	15	Peroxidase	[25]
Piperonyl butoxide (PiBx)	1.0 mM	15	NS CYP	[64]
Metyrapone	1.0 mM	5	NS CYP	[61]
Oleandomycin triacetate	0.5 mM	30	CYP3A1/2	[25]
Thio-tepa	0.1 mM	5	CYP2B1/2	[65]
Isoniazid	1.0 mM	5	CYP2E1	[25]
Diethyldithiocarbamate (DEDTCA)	0.1 mM	30	CYP2C > CYP2E1	[66]
Sulfaphenazole	0.1 mM	30	CYP2C	[66,67]

NS = nonselective.

## Abbreviations

ATCP	Aminotrichlorophenol
CYP	Cytochrome P450
DCNB	Dichloronitrobenzene
DTD	DT-diaphorase
FMO	Flavin monooxygenase
IRCC	Isolated renal cortical cells
LDH	Lactate dehydrogenase
MCNB	Monchloronitrobenzene
NAC	*N*-acetyl-l-cysteine
NR	Nitroreductase
PiBx	Piperonyl butoxide
ROS	Reactive oxygen species
TCA	Trichloroanilines
TCAA	Trichloroacetanilide
TCNB	Trichloronitrobenzene
TCNOP	Trichloronitrosobenzene
TCPHA	Trichlorophenylhydroxylamine
